# Allelic imbalance on chromosome 5q predicts long-term survival in neuroblastoma.

**DOI:** 10.1038/bjc.1996.645

**Published:** 1996-12

**Authors:** S. J. Meltzer, S. P. O'Doherty, C. N. Frantz, K. Smolinski, J. Yin, A. B. Cantor, J. Liu, M. Valentine, G. M. Brodeur, P. E. Berg

**Affiliations:** Department of Medicine, University of Maryland, Baltimore 21201, USA.

## Abstract

**Images:**


British Journal of Cancer (1996) 74, 1855-1861
© 1996 Stockton Press All rights reserved 0007-0920/96 $12.00

### Allelic imbalance on chromosome 5q predicts long-term survival in neuroblastoma

SJ Meltzer[1], SP O'Doherty[2], CN Frantz[2], K Smolinski[1], J Yin[1], AB Cantor[3], J Liu[2], M Valentine[4], GM Brodeur[5] and PE Berg[2]

[1]*Department of Medicine, G.I. Division, University of Maryland, BRB 8-007, 655 West Baltimore Street, Baltimore, MD 21201;* [2]*The Children's Cancer Foundation Research Laboratory, Department of Pediatrics, University of Maryland, BRB10-031, 655 West Baltimore Street, Baltimore, MD 21201;* [3]*Division of Cancer Control, Moffitt Cancer Center and Research Institute, 12902 Magnolia Drive, Tampa, FL 33612-9497;* [4]*Department of Experimental Oncology, St Jude's Children's Research Hospital, 332 North Lauderdale, Memphis, TN 38105;* [5]*Division of Oncology, The Children's Hospital of Philadelphia, 34th Street and Civic Center Boulevard, Philadelphia, PA 19104-4399, USA.*

**Summary** Neuroblastoma is the most common extracranial solid tumour of childhood. Amplification of the proto-oncogene, N-*myc*, confers a poor prognosis in neuroblastoma, while hyperdiploidy is associated with a favourable outcome. Little is known about the contribution of tumour-suppressor genes to the development or progression of neuroblastoma. We examined allelic imbalance at the locus of the tumour-suppressor gene, *APC* (adenomatous polyposis coli), on chromosome 5q using a polymerase chain reaction (PCR)-based assay. Nine of 24 (37.5%) informative neuroblastoma tumours showed allelic imbalance (AI) at this locus. Clinical data concerning N-*myc* amplification and DNA content were correlated with these results in the same patients. Allelic imbalance was found only in tumours containing a single copy of the N-*myc* gene and exhibiting hyperdiploidy. All nine patients with AI of chromosome 5q were alive after a median follow-up period of 46 months, while 7 of 15 (47%) of those lacking AI at this locus had died ($P=0.018$). Allelic imbalance at three additional loci on chromosome 5 was demonstrated in tumours that exhibited AI at the *APC* locus, suggesting that endoreduplication of chromosome 5 had occurred. Fluorescent *in situ* hybridisation (FISH) analysis of tumour tissue from one patient exhibiting AI demonstrated two, three, four or six copies of the *APC* gene per cell, consistent with this hypothesis. These data suggest that allelic imbalance of chromosome 5 is involved in at least a subset of neuroblastomas and influences survival in patients with neuroblastoma.

**Keywords:** neuroblastoma; polymerase chain reaction; allelic imbalance

Neuroblastoma is the most common extracranial solid tumour of childhood. It originates from primitive neural crest cells that form the sympathetic nervous sysem. Several clinical features of neuroblastoma suggest that discrete biological subgroups of this malignancy exist. Infants less than one year of age generally have a favourable response to therapy, and spontaneous regression is sometimes seen (Evans *et al.*, 1976; MacMillan *et al.*, 1976; Brodeur and Castleberry, 1993). Conversely, other neuroblastomas behave aggressively, often resulting in death despite surgery, high-dose chemoradiotherapy and autologous bone marrow transplantation. Determination of the molecular basis for these disparate clinical outcomes could improve our understanding of neuroblastoma and be valuable in planning therapy.

Several cytogenetic and molecular alterations have been described in neuroblastoma. In 1983, Schwab *et al.* determined that a gene termed N-*myc*, related to the proto-oncogene c-*myc*, was amplified in many human neuroblastoma cell lines (Schwab *et al.*, 1983). Subsequent studies have shown that DNA amplification of this gene, found in about 25% of neuroblastomas, is associated with rapid progression of disease (Seeger *et al.*, 1985; Look *et al.*, 1991; Fong *et al.*, 1992). In one study of neuroblastoma, DNA content, which grossly reflects chromosome number, was diploid (DNA index$=1$) in 44%, near-diploid (DNA index $\leqslant 1.18$) in 6%, near-triploid (DNA index$=1.25-1.68$) in 48% and hypotetraploid (DNA index$=1.85$) in 2% of 59 neuroblastoma tumours analysed (Bourhis *et al.*, 1991). In infants, hyperdiploidy is associated with a favourable outcome. Conversely, diploidy rarely occurs in localised disease, and it predicts a poor outcome in disseminated disease of infants (Look *et al.*, 1984, 1991).

No known tumour-suppressor genes have yet been directly associated with neuroblastoma, but published data suggest that tumour-suppressor gene loci may be important in this cancer. Loss of heterozygosity (LOH) has been demonstrated at chromosome 1p in 25%, at 11q in 42% and at 14q in 23% of neuroblastomas, while LOH was seen in only 5% of neuroblastomas following extensive studies of other loci (Brodeur *et al.*, 1977; Suzuki *et al.*, 1989; Srivatsan *et al.*, 1991; Fong *et al.*, 1992). Putative tumour-suppressor genes at 1p, 11q and 14q have not been identified. LOH at 1p is associated with a poor outcome in neuroblastoma, which may be caused at least in part by the observation that LOH of 1p frequently occurs in tumours with N-*myc* amplification (Fong *et al.*, 1989, 1992). It has been reported that the neurofibromatosis type I protein, which has tumour-suppressive properties, was absent in four of ten neuroblastoma cell lines (The *et al.*, 1993).

A number of tumour-suppressor genes have now been identified in other types of cancer. The most frequently affected tumour-suppressor gene is *p53*. LOH of 17p, the locus of the *p53* gene, was not seen in neuroblastoma (Imamura *et al.*, 1993; Komuro *et al.*, 1993; Vogan *et al.*, 1993). Another tumour-suppressor gene that has been associated with a variety of cancer types is *APC* (adenomatous polyposis coli), located on chromosome 5q21. This gene is responsible for the hereditary syndrome, familial adenomatous polyposis (Herrera *et al.*, 1986; Groden *et al.*, 1991; Joslyn *et al.*, 1991; Kinzler *et al.*, 1991; Nishisho *et al.*, 1991), and is involved by heterozygous deletion or mutation in sporadic colorectal cancers (Miyoshi *et al.*, 1992; Powell *et al.*, 1992), gastric carcinomas (Horii *et al.*, 1992*a*; Nakatsuru *et al.*, 1992), pancreatic cancers (Horii *et al.*, 1992*b*), oesophageal cancers (Boynton *et al.*, 1992) and in small-cell lung cancers (Ashton-Rickardt *et al.*, 1991; D'Amico *et al.*,

Correspondence: PE Berg
Received 27 February 1996; revised 20 August 1996; accepted 16 September 1996

1992). Because of the widespread involvement of the *APC* locus in a variety of cancers, we examined this genetic locus for allelic imbalance in neuroblastoma.

### Materials and methods

### *Patient samples*

Paired normal and tumour DNA samples were obtained from the Pediatric Oncology Group (POG) Neuroblastoma Tissue Bank. The samples were chosen to give a representative sampling of the full spectrum of neuroblastoma tumours based on patient age, stage and N-*myc* copy number. We also analysed clinical and tumour biological data on all informative cases. Information available included patient age, site of primary, stage, N-*myc* amplification, DNA index and survival. One child (POG number 105760) with stage Ds, diagnosed at age one month, was lost to follow up at 50 months and therefore was counted as censored at that time.

### *Polymerase chain reaction (PCR)*

*APC*-specific primers were used to amplify a 133 bp region of exon 11 of the *APC* gene using PCR (Meltzer *et al*., 1991). The primer sequences used were as follows: 5′-GGC TACATCTCCAAAAGTCAA-3′ and 5′-GGACTACAGGC-CATTGCAGAA-3′. The amplified segment contains a restriction site polymorphism for *Rsa*I at codon 486 of *APC*. In the presence of the restriction site, digestion of the PCR product with *Rsa*I yields 85 bp and 48 bp fragments. The PCR reaction contained 10 ng of genomic DNA and 1 unit of *Taq* polymerase (Promega, Madison, WI, USA) in a 50 $\mu$l reaction volume. The first PCR cycle was run at a denaturing temperature of 95°C for 105 s, an annealing temperature of 59°C for 90 s and a polymerisation temperature of 72°C for 45 s. The subsequent 28 cycles were 20 s at 95°C, 45 s at 59°C and 30 s at 72°C. A control tube that contained no genomic DNA was included in each experiment.

The entire PCR product was digested overnight with 30 units of *Rsa*I in a reaction volume of 60 $\mu$l. A PCR product that was known to be homozygous for the *Rsa*I site was used to verify complete digestion each time a digestion reaction was performed. The products of digestion were electrophoresed on a 7% native polyacrylamide gel containing 5% glycerol at 150 V for 90 min. The gel was then stained with ethidium bromide, destained and photographed using Polaroid 55 negative/positive film. A laser densitometer (LKB, Bromma, Sweden) and Gel Scan XL computer program (Bromma, Sweden) were used to quantitate bands on the Polaroid negatives. Our data readings were within the linear capabilities of the equipment used.

The data obtained from the laser densitometer was determined to be reproducible by repeated amplification and cleavage of normal DNA from three heterozygotes followed by electrophoresis and multiple scans of the negatives (data not shown). In order to determine the linear range of PCR amplification of the *APC* fragment, DNA samples were amplified for 24, 26, 28, 30 and 32 cycles as described above. The PCR product reached a plateau between 30 and 32 PCR cycles (data not shown); therefore, 28 cycles were used to maximise PCR product, while remaining in the linear range of amplification.

Theoretically, a mismatch of DNA strands in the final cycle of the PCR reaction could lead to heterodimer formation, that is, annealing of single-stranded DNA containing an *Rsa*I site to single-stranded DNA lacking the site. In the presence of a heterodimer, *Rsa*I would fail to cut despite the presence of the restriction site motif on one DNA strand, thus artifactually increasing the measured uncut allele. We therefore performed in triplicate a mixing experiment in which known amounts of genomic DNA homozygous for the presence or absence of the *Rsa*I site were mixed before PCR amplification. These samples were then analysed as described previously. The results showed that there was no heterodimer formation, since the percentage of cut and uncut alleles after PCR was the same as the percentage of each template added to the PCR reaction (data not shown). These results confirmed those of a similar mixing experiment (Meltzer *et al*., 1991).

Normal DNA from patients' blood was PCR amplified, digested, electrophoresed, photographed and scanned using a laser densitometer. Patients whose normal DNA was informative were studied further by comparing their normal and tumour DNA. To determine which tumours showed allelic imbalance, we used a combination of visual inspection of the polyacrylamide gel, analysis of the allele index ratio (described below) and three types of statistical cluster analyses of the data obtained from scanning the gel (SAS Institute, 1989). Estimates of survival curves were produced using the method of Kaplan and Meier (Kaplan and Meier, 1958). Standard errors of these estimates were obtained by Peto's formula (Peto *et al*., 1977). Survival curves were compared using the two-sided log-rank test (Mantel, 1968).

### *Other 5q loci*

In cases showing AI involving the *APC* gene, loci D5S471 (5q23), D5S484 (5q15) and D5S623 (5p12) were also amplified using primers and protocols from Research Genetics (Huntsville, AL, USA). Thus, PCR amplification on both telomeric and centromeric sides of *APC*, as well as on the short arm of chromosome 5, were performed.

### *Allele index ratio*

The allele index ratio was calculated by dividing the densitometer reading of the uncut allele by the sum of the two cut alleles. In normal DNA from heterozygotes, in which both alleles were present in equal amounts, the mean allele index ratio was $1.06 \pm 0.10$ in 24 samples scanned. Tumours that showed apparent allelic imbalance were PCR amplified and analysed independently at least three times.

### *Fluorescence* in situ *hybridisation (FISH)*

Purified DNA from a genomic clone representing the *APC* locus was labelled with digoxigenin dUTP by nick translation. Labelled probe was combined with sheared human DNA and hybridised to touch preps made from neuroblastoma cells in a solution containing 50% formamide, 10% dextran sulphate and 2 × saline sodium citrate (SSC). Specific hybridisation signals were detected by incubating the hybridised slides in fluorescein-conjugated anti-digoxigenin antibodies.

### Results

We studied 42 paired samples of normal and neuroblastoma tumour DNAs for LOH of chromosome 5q at the *APC* gene locus. Informative samples, heterozygous for the *Rsa*I restriction site, exhibited three distinct bands: 133 bp from the uncut allele, and 85 bp and 48 bp from the cut allele. Uninformative samples contained either two bands, 85 bp and 48 bp in length, or a single band of 133 bp depending on the presence or absence of the *Rsa*I site respectively. Of the 42 samples, 24 (57%) were informative for the *Rsa*I restriction site.

Among the 24 informative samples, we observed an imbalance of the two alleles, rather than complete loss of heterozygosity. To determine quantitatively which tumour DNAs exhibited allelic imbalance (AI), an allele index ratio (AIR) was calculated (see Materials and methods), with the results shown in Table I. In each case, the AIR was approximately 1.0 for the normal DNA samples (data not shown), but varied widely for the tumour samples. A histogram showing the frequency distribution of the AIR in normal and tumour samples is presented in Figure 1. In the

**Table I** Clinical and molecular biological characteristics of tumours

| *Sample number* | *Allelic index ratio* | *N*-myc *number* | *DNA index* | *Clinical stage* | *Follow up time (months)*[a] |
|---|---|---|---|---|---|
| A. Patients exhibiting allelic imbalance of chromosome 5q | | | | | |
| 20 | 0.69 | 1 | 1.33 | Ds | 51 (L) |
| 150 | 1.69 | 1 | 1.19 | C | 69 (A) |
| 254 | 0.68 | 1 | NA[b] | B | 51 (A) |
| 287 | 1.76 | 1 | 1.46 | C | 54 (A) |
| 368 | 1.53 | 1 | NA | A | 46 (A) |
| 539 | 1.99 | 1 | 1.47 | C | 35 (A) |
| 556 | 1.74 | 1 | 1.55 | C | 30 (A) |
| 580 | 0.69 | 1 | 1.20 | A | 33 (A) |
| 897 | 0.69 | 1 | 1.29 | A | 12 (A) |
| B. Patients not exhibiting allelic imbalance of chromosome 5q | | | | | |
| 13 | 1.13 | 1 | NA | A | 93 (A) |
| 34 | 0.98 | 200 | 1.00 | C | 17 (D) |
| 35 | 0.99 | 10 | 1.77 | C | 10 (D) |
| 326 | 0.98 | 50 | 1.19 | D | 0.2 (D) |
| 353 | 1.09 | 1 | 1.30 | C | 4 (A) |
| 594 | 1.04 | 1 | 1.00 | Ds | 33 (A) |
| 649 | 1.08 | 150 | 1.00 | C | 21 (D) |
| 651 | 1.01 | 1 | 1.09 | D | 28 (D) |
| 661 | 0.96 | 1 | NA | C | 18 (A) |
| 691 | 0.92 | 1 | 1.23 | B | 26 (A) |
| 701 | 1.16 | 1 | 1.22 | Ds | 23 (A) |
| 714 | 1.21 | 1 | 1.90 | Ds | 1 (D) |
| 747 | 0.97 | 75 | 1.00 | D | 125 (A) |
| 759 | 1.03 | 1 | 1.86 | A | 25 (A) |
| 767 | 1.31 | ND[b] | 1.00 | D | 6 (D) |

[b]ND, not determined; NA, not available. [a]A, alive; L, lost to follow up; D, dead.

**Figure 1** Frequency distribution of the allele index (ratio of uncut/cut allele) in normal (**a**) and tumour (**b**) samples.

**Figure 2** Allelic imbalance at the *APC* locus. The position of the band from the uncut allele (133 bp) and two bands from the cut allele (85 and 48 bp) are indicated by arrows. Normal and tumour DNA from the same patient are displayed side by side. DNA from blood and the corresponding tumour DNA from patient 691 after amplification and *Rsa*I digestion are shown in lanes 1 and 2 respectively. In this patient, there was an equal proportion of uncut (upper 133 bp band) to cut (lower bands 85 and 48 bp) alleles following digestion, therefore no allelic imbalance was present. Lanes 3 and 4 show DNA from the blood and tumour, respectively, of patient 539 exhibiting allelic imbalance of the cut allele.

tumour samples, there were three distinct groups: one had an AIR of approximately 1.0, which was within the normal range; the second group had an AIR of greater than 1.5, indicating reduction of the cut allele; and the third group had an AIR of less than 0.7, indicating reduction of the uncut allele. Nine of the 24 (37.5%) informative tumour DNAs exhibited allelic imbalance of chromosome 5q. Allelic imbalance was approximately equally distributed between the two alleles, with five of nine (55%) showing loss of the cut allele and four of nine (45%) showing loss of the uncut allele. Representative DNA samples from two patients are shown in in Figure 2.

Clinical data were available for each of the patients we analysed (Table I). This incuded age at diagnosis, site of primary tumour, clinical stage, N-*myc* copy number, DNA ploidy, follow-up interval and survival. Using Wilcoxon's rank sum test for age and Fisher's exact test to compare characteristics of those patients with and without AI with respect to these parameters, except for survival, we determined that all two-sided *P*-values exceeded 0.10 (data not shown). Because of the small sample sizes, we cannot exclude the possibility that these groups actually do differ on some of these characteristics. The status of chromosome 1p was unavailable for the majority of patients.

Among the patients exhibiting AI, there were no POG stage D patients (Hayes *et al.*, 1983; Hayes and Smith, 1989). In contrast, there were four stage D patients among the 15 patients lacking AI, suggesting a possible association between the presence of AI and less severe disease. In fact, all nine patients are alive after a median follow-up period of 46 months, while seven (47%) of those without AI have died. From the Kaplan–Meier lifetime probability curves, three-year survival of patients without AI of 5q was estimated to be 49% (data not shown). The improved survival associated with AI of 5q was statistically significant ($P = 0.018$).

To determine the background rate of allelic imbalance, we examined the loci of two additional tumour-suppressor genes, *p53* on chromosome 17 and *DCC* (deleted in colorectal cancer) on chromosome 18 (Fearon *et al.*, 1990) (Table II). DNA from the same 42 patients was amplified by PCR using primers specific for a variable number of tandem repeats (VNTR) within *p53* or primers specific for a polymorphic site in the *DCC* gene, and the products were analysed. For *p53*, 26 cases were informative, of which three exhibited AI (11.5%). One of them (patient 150) also exhibited AI at the *APC* locus, one (patient 606) was not informative at *APC* and the third (patient 701) did not exhibit AI on chromosome 5q. One additional patient (patient 767) exhibited complete loss of one allele, demonstrating that our PCR-based technique can distinguish between LOH and AI. Of the 27 informative cases for the *DCC* locus, only one (3.7%) exhibited allelic imbalance; this case was not informative at the *APC* locus.

Since the cells or nuclei from tumours were not sorted or otherwise purified, contamination of tumour tissue with normal tissue may have contributed to the fact that we observed AI rather than LOH. To investigate this possibility, nuclei from tumour tissue of patient 254 were flow cytometrically sorted, then re-examined by PCR after separation of hyperdiploid nuclei from normal, diploid

**Table II** Allelic imbalance on chromosomes 17 and 18

| *Chromosome* | *Sample number* | *N*-myc *number* | *DNA index* | *Clinical stage* | *Follow-up time (months)*[a] | *APC status* |
|---|---|---|---|---|---|---|
| 17 | 150 | 1 | 1.19 | C | 69 (A) | AI |
| | 606 | 1 | 1.82 | D | 29 (D) | NI |
| | 701 | 1 | 1.22 | Ds | 23 (A) | Negative |
| 18 | 675 | 1 | 1.34 | D | 15 (A) | NI |

[a]A, alive; L, lost to follow-up; D, dead. NI, not informative.

**Figure 3** FISH analysis of the *APC* locus.

nuclei. The AI of DNA from hyperdiploid nuclei was unchanged (i.e., still imbalanced) after sorting, while the AI of DNA from the diploid nuclei was in the normal range (data not shown).

In six of the cases exhibiting AI, we examined markers D5S471 (5q23), D5S484 (5q15) and D5S623 (5p12). Where results were informative, AI was also seen at these loci (data not shown).

Finally, to determine whether the AI we observed resulted from an extra copy of part or all of chromosome 5, FISH was performed on tumour tissue from patient 254, the only patient for whom tissue was available. The probe was a cosmid containing the genomic *APC* gene. Analysis of this experiment revealed that the cells contained two, three, four or six copies of the *APC* gene per cell (Figure 3). Of 200 cells analysed, 43% exhibited two copies, 26% exhibited three copies, 22% exhibited four copies and 9% exhibited six copies per cell. No single chromosome contained more than one copy of the *APC* gene, suggesting that endoreduplication of part of chromosome 5 had not occurred in this patient. Since all cells contained at least two copies of *APC*, it is unlikely that a deletion occurred. The presence of three, four or six copies is consistent with hyperdiploidy, although the DNA index for this patient is not known.

### Discussion

We have shown that AI involving *APC* and other loci on chromosome 5 occurs in 37.5% of neuroblastomas. Since AI occurred in only 11.5% of informative cases at the *p53* locus and 3.7% of informative cases at the *DCC* locus in our study, as well as at comparably low rates for other loci in other studies (Brodeur *et al.*, 1977; Suzuki *et al.*, 1989; Srivatsan *et al.*, 1991; Fong *et al.*, 1992), our data for chromosome 5 are well above background for neuroblastomas. These data imply that the entire chromosome 5 was involved in the allelic imbalance we initially observed at the *APC* locus. Interestingly, AI on chromosome 5 locus tends to occur in a subset of neuroblastoma tumours in which the N-*myc* gene is not amplified, the DNA index is greater than 1.00 and survival is excellent.

Although the detection of AI was consistent and reproducible, we did not observe complete loss of one allele. There are several possible explanations for this observation: (1) Contamination of tumour tissue with normal diploid tissue may have occurred, since the nuclei were not sorted. This is unlikely because, in tumour tissue of patient 254, DNA from hyperdiploid nuclei still exhibited the same AIR as the unsorted sample, while DNA from diploid nuclei did not show allelic imbalance; (2) The tumour cell preparations may have included a mixture of some tumour cells retaining and some lacking chromosome 5. This could have been due to polyclonality within the tumour or to the continued progression of neuroblastoma, with AI of chromosome 5 constituting a late event; (3) A likely possibility is that we may have been observing endoreduplication of one homologue of chromosome 5, consistent with the fact that all of the cases exhibiting AI for whom data were available were hyperdiploid. This hypothesis is supported by FISH analysis of tumour tissue from one patient exhibiting AI, in which there were two, three, four, or six copies of the *APC* gene.

The AI we observed was widespread and involved multiple loci on both arms of chromosome 5, suggesting reduplication of the entire chromosome or large portions of it. Endoreduplication would lead to an apparent increase in either the cut or uncut allele in tumour DNA compared with normal DNA, as we observed. Indeed, an extra chromosome 5 has been noted to occur in several neuroblastoma karyotypes (Kaneko *et al.*, 1987; Hayashi *et al.*, 1989; Look *et al.*, 1991).

The presence of additional alleles of *APC* or another gene(s) on chromosome 5 may affect tumorigenesis and tumour behaviour. In other studies, *APC* copy number was found to influence survival (Shimada and Masayuki, 1994). An intriguing possibility in neuroblastoma is that additional wild-type *APC* alleles present in tumour cells may have suppressed their growth rate and/or tumorigenicity via a dominant negative or 'threshold' effect. Precedent exists for the dominant negative phenomenon in the case of *p53*, in which introduction of a single copy of the wild-type gene into osteosarcoma cells containing an endogenous mutated *p53* gene suppressed the tumorigenicity of these cells and slowed their growth rate (Chen *et al.*, 1990). Similarly, introduction of one copy of the *p53* gene into a human lung carcinoma cell line containing two normal *p53* alleles also caused a decreased growth rate and a decreased proportion of S-phase cells (Noble *et al.*, 1992). An alternative theory of tumour-suppressor gene inactivation is the 'threshold hypothesis', which holds that a critical amount of the gene product is necessary for proper gene function. This theory is supported by studies of melanoma cells, in which the degree of growth suppression was proportional to the number of copies of chromosome 6 that were introduced into cells (Trent *et al.*, 1990; Robertson *et al.*, 1996). By analogy, extra copies of chromosome 5 may confer growth-suppressive effects on neuroblastoma cells and an advantage to patients, consistent with the favourable prognosis we observed for 5q allelic imbalance and already associated with hyperdiploidy in neuroblastoma.

### Acknowledgements

We wish to thank Dr Bryan J Reid, University of Washington, Seattle, WA, USA, for sorting nuclei and extracting DNA from the sorted nuclei, and Dr Akira Horii for providing the genomic clone of the *APC* gene. This work was supported in part by grants from the Pediatric Oncology Group NCI/NIH Grant CA 30969 (PEB), American Cancer Society Grant 93-01 (PEB), Children's Cancer Foundation (PEB and CNF), National Research Service Award CA 09633 (SOD), NCI/NIH CA 29139 (ABC), NIH DK47717 (SJM), NIH ES 07120 (SJM), NASA 9307-0502 (SJM), NIH CA 7497 (SJM), ACS EDT-37A (SJM) and the Office of Medical Research, Department of Veterans Affairs (SJM).

### References

ASHTON-RICKARDT PG, WYLLIE AH, BIRD CC, DUNLOP MG, STEEL CM, MORRIS RG, PIRIS J, ROMANWOSKI P, WOOD R, WHITE R AND NAKAMURA Y. (1991). MCC, a candidate familial polypolis gene in 5q21, shows frequent allele loss in colorectal and lung cancer. *Oncogene*, **6**, 1881–1886.

BOURHIS J, DEVATHAIRE F, WILSON GD, HARTMANN O, TERRIER-LACOMBE MJ, BOCCON-GIBOD L, MCNALLY NJ, LEMERLE J, RIOU G AND BERNARD J. (1991). Combined analysis of DNA ploidy index and N-*myc* genomic content in neuroblastoma. *Cancer Res.*, **51**, 33–36.

BOYNTON RF, BLOUNT PL, YIN J, BROWN VL, HUANG Y, TONG Y, MCDANIEL T, NEWKIRK C, RESAU JH, RASKIND WH, HAGGITT RC, REID BJ AND MELTZER SJ. (1992). Loss of heterozygosity involving the APC and MCC genetic loci occurs in the majority of human esophageal cancers. *Proc. Natl Acad. Sci. USA*, **89**, 3385–3388.

BRODEUR GM AND CASTLEBERRY RP. (1993). Neuroblastoma. In *Principles and Practice of Pediatric Oncology*, Pizzo PA and Poplack DG (eds) pp. 739–768. Lippincott: Philadelphia.

BRODEUR GM, SEKHON GS AND GOLDSTEIN MN. (1977). Chromosomal aberations in human neuroblastomas. *Cancer*, **40**, 2256–2263.

CHEN P-L, CHEN Y, BOOKSTEIN R AND LEE W-H. (1990). Genetic mechanisms of tumor suppression by the human p53 gene. *Science*, **250**, 1576–1580.

D'AMICO D, CARBONE DP, JOHNSON BE, MELTZER SJ AND MINNA JD. (1992). Polymorphic sites within the MCC and APC loci reveal very frequent loss of heterozygosity in human small cell lung cancer. *Cancer Res.*, **52**, 1996–1999.

EVANS AE, GERSON J AND RANDOLPH J. (1976). Spontaneous regression of neuroblastoma. *Natl Cancer Inst. Monogr.*, **44**, 49–53.

FEARON ER, CHO KR, NIGRO JM, KERN SE, SIMONS JW, RUPPERT JM, HAMILTON SR, PREISINGER AC, THOMAS G, KINZLER KW AND VOGELSTEIN B. (1990). Identification of a chromosome 18q gene that is altered in colorectal cancers. *Science*, **247**, 49–56.

FONG C-T, DRACOPOLI ND, WHITE PS, MERRILL PT, GRIFFITH RC, HOUSMAN DE AND BRODEUR GM. (1989). Loss of heterozygosity for chromosome 1p in human neuroblastomas: correlation with N-*myc* amplification. *Proc. Natl Acad. Sci. USA*, **86**, 3753–3757.

FONG C, WHITE PS, PETERSON K, SAPIENZA C, CAVENEE WK, KERN S, VOGELSTEIN B, CANTOR AB, LOOK AT AND BRODEUR GM. (1992). Loss of heterozygosity for chromosome 1 or 14 defines subsets of advanced neuroblastomas. *Cancer Res.*, **52**, 1780–1785.

GRODEN J, THILVERIS A, SAMOWITZ W, CARLSON M, GELBERT L, ALBERTSEN H, JOSLYN G, STEVENS J, SPIRIO L, ROBERTSON M, SARGEANT L, KRAPCHO K, WOLFF E, BURT R, HUGHES JP, WARRINGTON J, McPHERSON J, WASMUTH J, LEPASLIER D, ABDERRAHIM H, COHEN D, LEPPERT M AND WHITE R. (1991). Identification and characterization of the familial adenomatous polyposis coli gene. *Cell*, **66**, 589–600.

HAYASHI Y, KANDA N, INABA T, HANADA R, NAGAHARA N, MUCHI H AND YAMAMOTO K. (1989). Cytogenetic findings and prognosis in neuroblastoma with emphasis on marker chromosome 1. *Cancer*, **63**, 126–132.

HAYES FA AND SMITH EI. (1989). Neuroblastoma. In *Principles and Practice of Pediatric Oncology*, Pizzo PA and Poplack DG (eds) pp. 614–615. Lippincott: Philadelphia.

HAYES FA, GREEN A, HUSTU H AND KUMAR M. (1983). Surgicopathological staging of neuroblastoma: prognostic signficance of regional lymph node metastases. *J. Pediat.*, **102**, 59–62.

HERRERA L, KAKATI S, GIBAS L, PIETRZAK E AND SANDBERG A. (1986). A brief clinical report: Gardner syndrome in a man with an interstitial deletion of 5q. *Am. J. Med. Genet.*, **25**, 47476–47483.

HORII A, NAKATSURU S, MIYOSHI Y, ICHII S, NAGASE H, KATO Y, YANAGISAWA A AND NAKAMURA Y. (1992*a*). The APC gene, responsible for familial adenomatous polyposis, is mutated in human gastric cancer. *Cancer Res.*, **52**, 3231–3233.

HORII A, NAKATSURU S, MIYOSHI Y, ICHII S, NAGASE H, ANDO H, YANAGISAWA A, TSUCHIYA E, KATO Y AND NAKAMURA Y. (1992*b*). Frequent somatic mutations of the APC gene in human pancreatic cancer. *Cancer Res.*, **52**, 6696–6698.

IMAMURA J, BARTRAM CR, BERTHOLD F, HARMS D, NAKAMURA H AND KOEFFLER HP. (1993). Mutation of the p53 gene in neuroblastoma and its relationship with N-*myc* amplification. *Cancer Res.*, **53**, 4053–4058.

JOSLYN G, CARLSON M, THILVERIS A, ALBERTSEN H, GELBERT L, SAMOWITZ W, GRODEN J, STEVENS J, SPIRIO L, ROBERTSON M, SARGEANT L, KRAPCHO K, WOLFF E, BURT R, HUGHES JP, WARRINGTON J, McPHERSON J, WASMUTH J, LEPASLIER D, ABDERRAHIM H, COHEN D, LEPPERT M AND WHITE R. (1991). Identification of deletion mutations and three new genes at the familial polyposis locus. *Cell*, **66**, 601–613.

KANEKO Y, KAND N, MASEKI N, SAKURAI M, TSUCHIDA Y, TAKEDA T, OKABE I AND SAKURAI M. (1987). Different karyotypic patterns in early and advanced stage neuroblastomas. *Cancer Res.*, **47**, 311–318.

KAPLAN EL AND MEIER P. (1958). Nonparametric estimation from incomplete observation. *J. Am. Stat. Assoc.*, **53**, 457–481.

KINZLER KW, NILBERT MD, SU L-K, VOGELSTEIN B, BRYAN TM, LEVY DB, SMITH KJ, PREISINGER AC, HEDGE P, McKECHNIE D, FINNIEAR R, MARKHAM A, GROFFEN J, BOGUSKI MS, ALTSCHUL SF, HORII A, ANDO H, MIYOSHI Y, MIKI Y, NISHISHO I AND NAKAMURA Y. (1991). Identification of FAP locus genes from chromosome 5q21. *Science*, **253**, 661–665.

KOMURO H, HAYASHI Y, KAWAMURA M, HAYASHI K, KANEKO Y, KAMOSHITA S, HANADA R, YAMAMOTO I, HONGO T, YAMADA M AND TSUCHIDA Y. (1993). Mutations of the p53 gene are involved in Ewing's sarcoma but not in neuroblastomas. *Cancer Res.*, **53**, 5284–5288.

LOOK AT, HAYES FA, NITSCHKE R, McWILLIAMS NB AND GREEN AA. (1984). Cellular DNA content as a predictor of response to chemotherapy in infants with unresectable neuroblastoma. *N. Engl. J. Med.*, **311**, 231–235.

LOOK AT, HAYES A, SHUSTER JJ, DOUGLASS EC, CASTLEBERRY RP, BOWMAN LC, SMITH EI AND BRODEUR GM. (1991). Clinical relevance of tumor cell ploidy and N-*myc* gene amplification in childhood neuroblastoma. *J. Clin. Oncol.*, **9**, 581–591.

MACMILLAN RW, BLANC WB AND SANTULLI TV. (1976). Maturation of neuroblastoma to ganglioneroma in lymph nodes. *J. Pediatr. Surg.*, **11**, 461–462.

MANTEL N. (1968). Evaluation of survival data and two new rank order statistics arising in its consideration. *Cancer Chemother. Rep.*, **68**, 163–170.

MELTZER SJ, YIN J, HUANG Y, McDANIEL T, NEWKIRK C, ISERI O, VOGELSTEIN B AND RESAU JH. (1991). Reduction to homozygosity involving p53 in esophageal cancers demonstrated by the polymerase chain reaction. *Proc. Natl Acad. Sci. USA*, **82**, 7575–7579.

MIYOSHI Y, NAGASE H, ANDO H, HORII A, ICHII S, NAKATSURU S, AOKI T, MIKI Y, MORI T AND NAKAMURA Y. (1992). Somatic mutations of the APC gene in colorectal tumors: mutation cluster region in the APC gene. *Hum. Mol. Genet.*, **1**, 229–233.

NAKATSURU S, YANAGISAWA A, ICHII S, TAHARA E, KATO Y, NAKAMURA Y AND HORII A. (1992). Somatic mutation of the APC gene in gastric cancer: frequent mutations in very well differentiated adenocarcinoma and signet-ring carcinoma. *Hum. Mol. Genet.*, **1**, 559–563.

NISHISHO I, NAKAMURA Y, MIYOSHI Y, MIKI Y, ANDO H, HORII A, KOYAMA K, UTSONOMYA J, BABA S, HEDGE P, MARKHAM A, KRUSH AJ, PETERSEN G, HAMILTON SR, NILBERT MC, LEVY DB, BRYAN TM, PRESINGER AC, SMITH KJ, SU L, KINZLER KW AND VOGELSTEIN B. (1991). Mutations of chromosome 5q21 genes in FAP and colorectal cancer patients. *Science*, **253**, 665–669.

NOBLE JR, WILLETTS KE, MERCER WE AND REDDEL RR. (1992). Effects of exogenous wild-type p53 on a human lung carcinoma cell line with endogenous wild-type p53. *Exp. Cell Res.*, **203**, 297–304.

PETO R, PIKE MC, ARMITAGE P, BRESLOW NE, COX DR, HOWARD SV, MANTEL N, McPHERSON K, PETO J AND SMITH PG. (1977). Design and analysis of randomized clinical trials. Part II: analysis and examples. *Br. J. Cancer*, **35**, 1–39.

POWELL SM, ZILZ N, BEAZER-BARCLAY Y, BRYAN TM, HAMILTON SR, THIBODEAU SN, VOGELSTEIN B AND KINZLER KW. (1992). APC mutations occur early during colorectal tumorigenesis. *Nature*, **359**, 235–237.

ROBERTSON GP, COLEMAN AB AND LUGO TG. (1996). Mechanisms of human melanoma cell growth and tumor suppression by chromosome 6. *Cancer Res.*, **56**, 1635–1641.

SAS INSTITUTE, INC. (1989). The CLUSTER Procedure. In *SAS/STAT User's Guide*, Version 6, Fourth Edition, Volume 1, pp. 519–614. Cary, NC, USA.

SCHWAB M, ALITALO K, KLEMPNAUER K-H, VARMUS HE, BISHOP JM, GILBERT F, BRODEUR G, GOLDSTEIN M AND TRENT J. (1983). Amplified DNA with limited homology to *myc* cellular oncogene is shared by human neuroblastoma cell lines and a neuroblastoma tumor. *Nature*, **305**, 245–248.

SEEGER RC, BRODEUR GM, SATHER H, DALTON A, SIEGEL S, WONG KY AND HAMMOND D. (1985). Association of multiple copies of the N-*myc* oncogene with rapid progression of neuroblastomas. *N. Engl. J. Med.*, **313**, 1111–1116.

SHIMADA Y AND MASAYUKI I. (1994). Primary cell culture of esophageal cancer as an indicator of biological malignancy. *Hum. Cell*, **7**, 193–198.

SRIVATSAN ES, MURALI V AND SEEGER RC. (1991). Loss of heterozygosity for alleles on chromosome 1q and 14q in neuroblastoma. *Prog. Clin. Biol. Res.*, **366**, 91–98.

SUZUKI T, YOKOTA J, HIDEO M, OKABE I, OOKUNI M, SUGIMIRA T AND TERADA M. (1989). Frequent loss of heterozygosity on chromosome 14q in neuroblastoma. *Cancer Res.*, **49**, 1095–1098.

THE I, MURTHY AE, HANNIGAN GE, JACOBY LB, MENON AG, GUSELLA JF AND BERNARDS A. (1993). Neurofibromatosis type 1 gene mutations in neuroblastoma. *Nature Genet.*, **3**, 62–66.

TRENT JM, STANBRIDGE EJ, MCBRIDE HL, MEESE EU, CASEY G, ARAUJO DE, WITKOWSKI CM AND NAGLE RB. (1990). Tumorigenicity in human melanoma cell lines controlled by introduction of human chromosome 6. *Science*, **247,** 568–571.

VOGAN K, BERNSTEIN M, LECLER J-M, BRISSON L, BROSSARD J, BRODEUR GM, PELLETIER J AND GROS P. (1993). Absence of p53 mutations in primary neuroblastomas. *Cancer Res.*, **53,** 5269–5273.


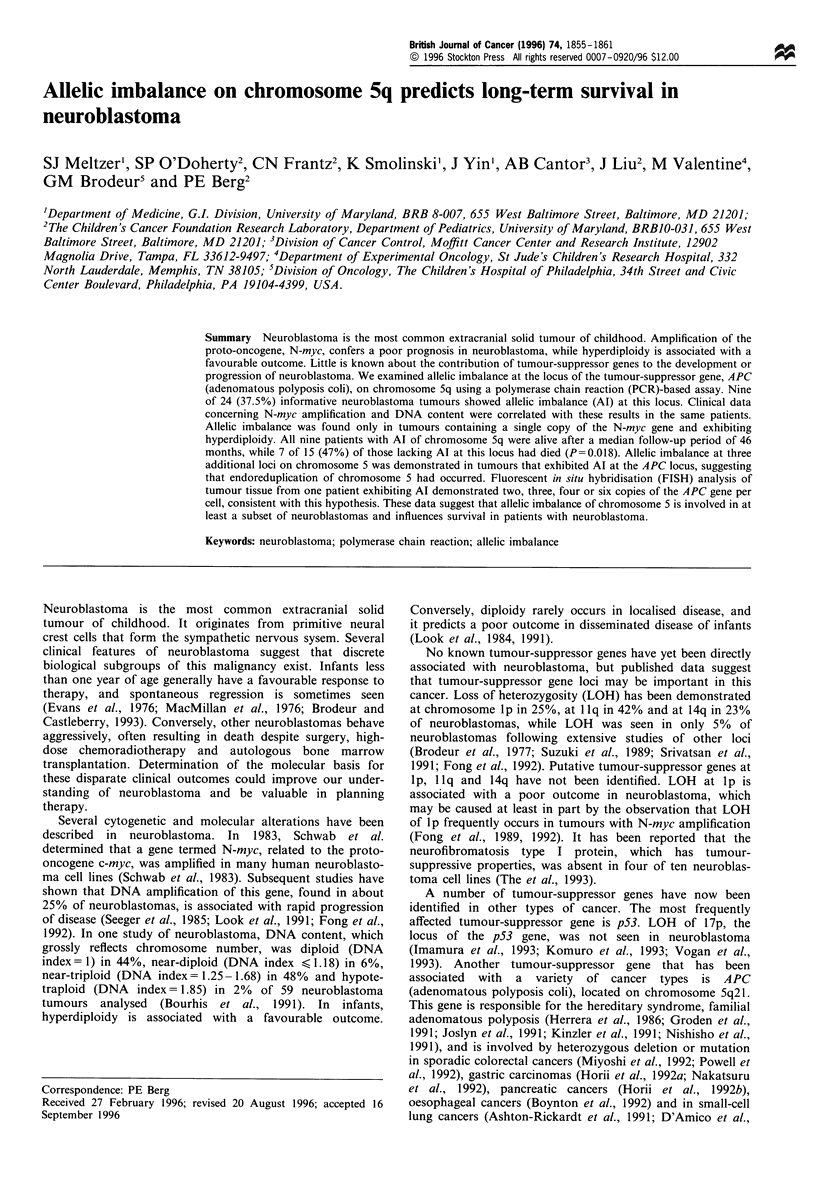

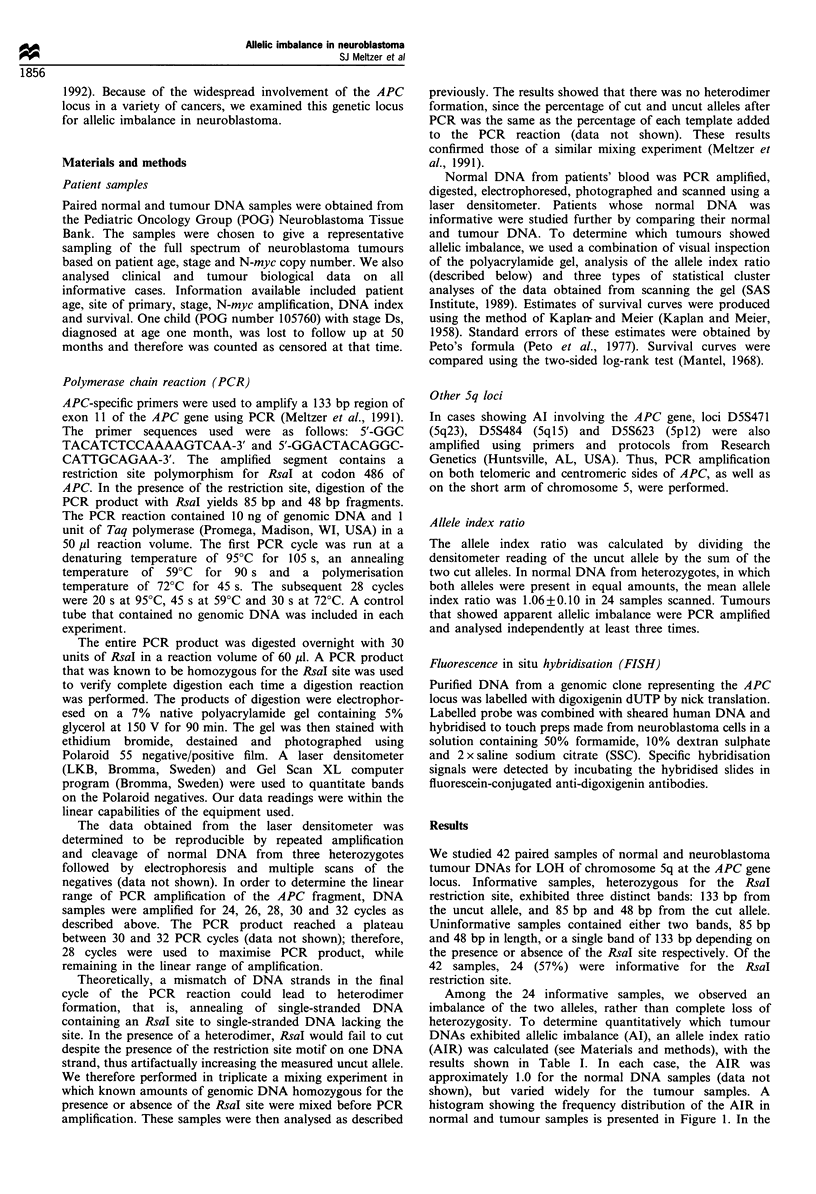

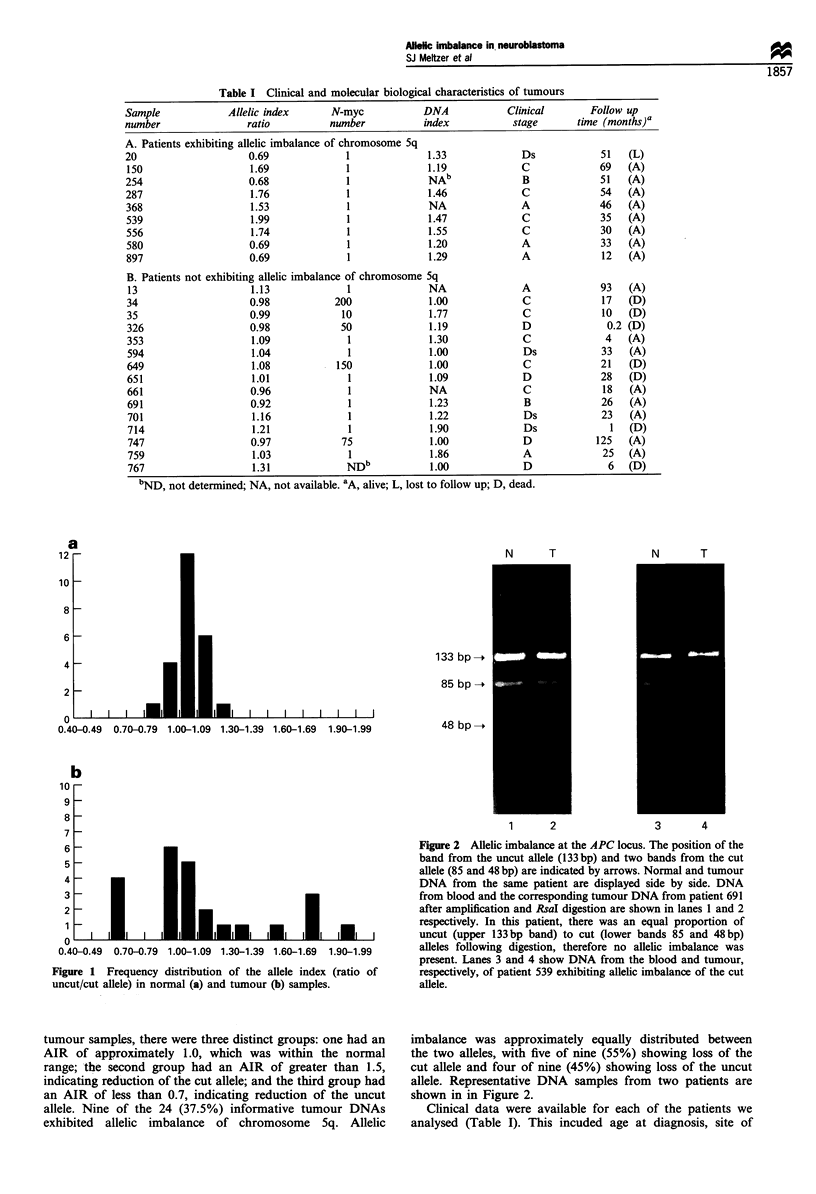

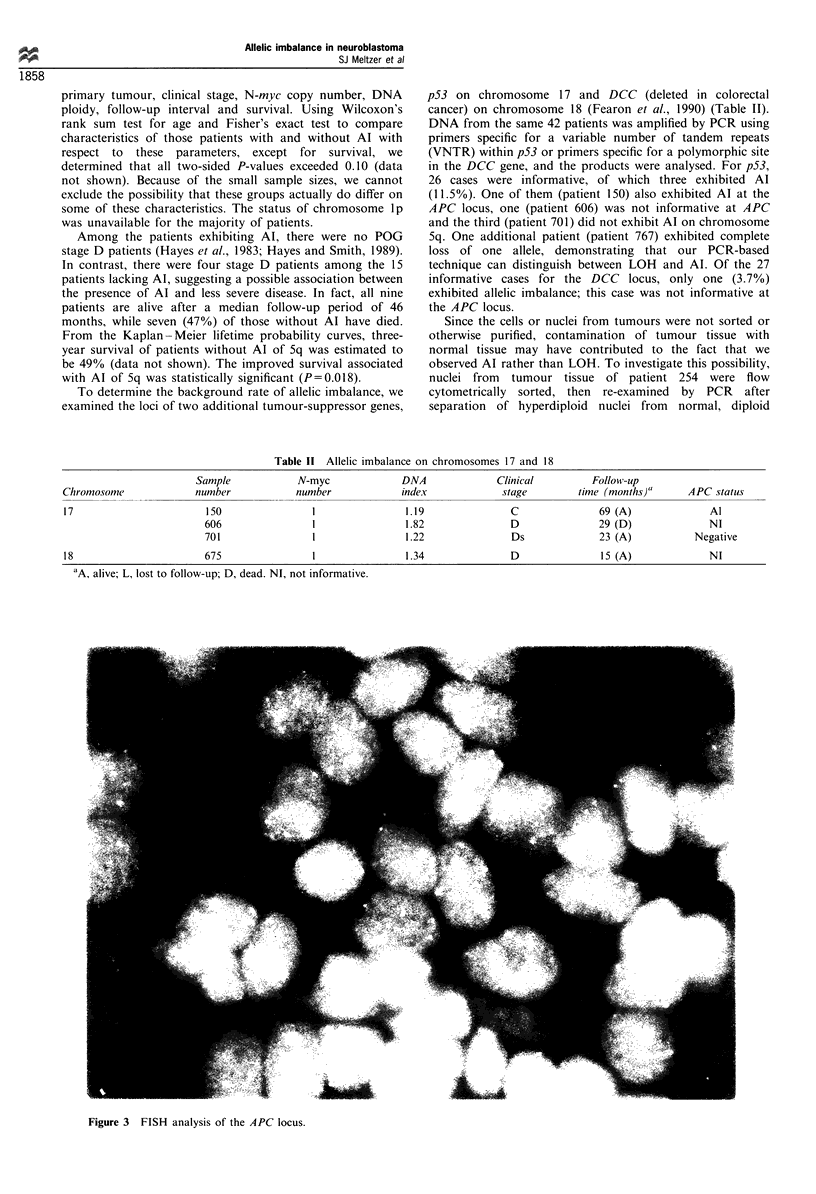

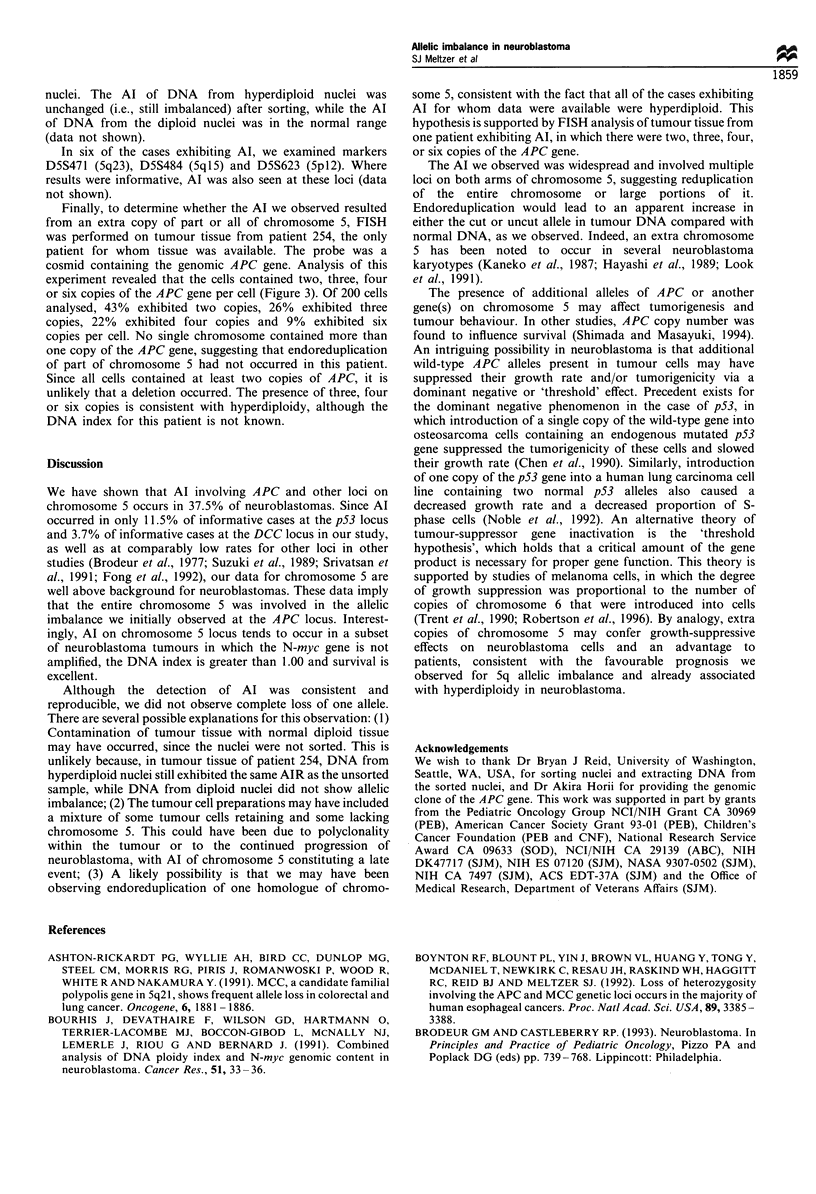

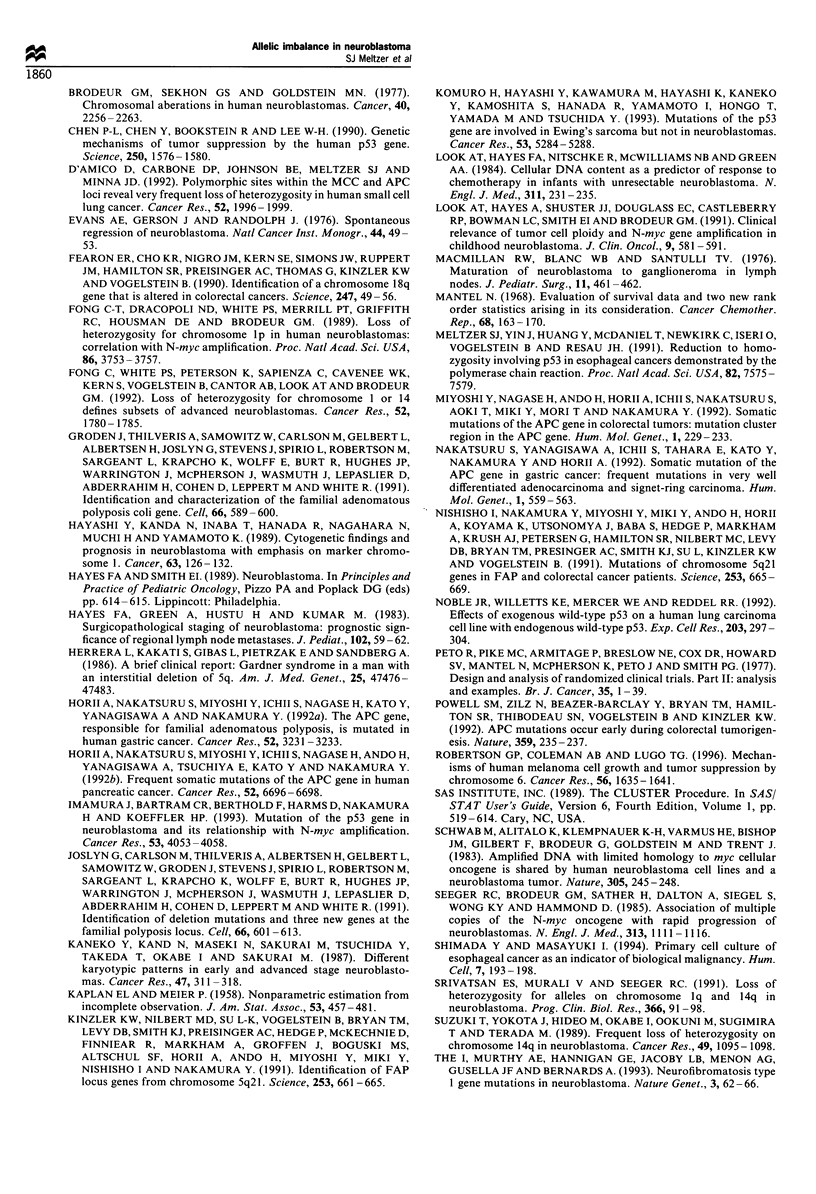

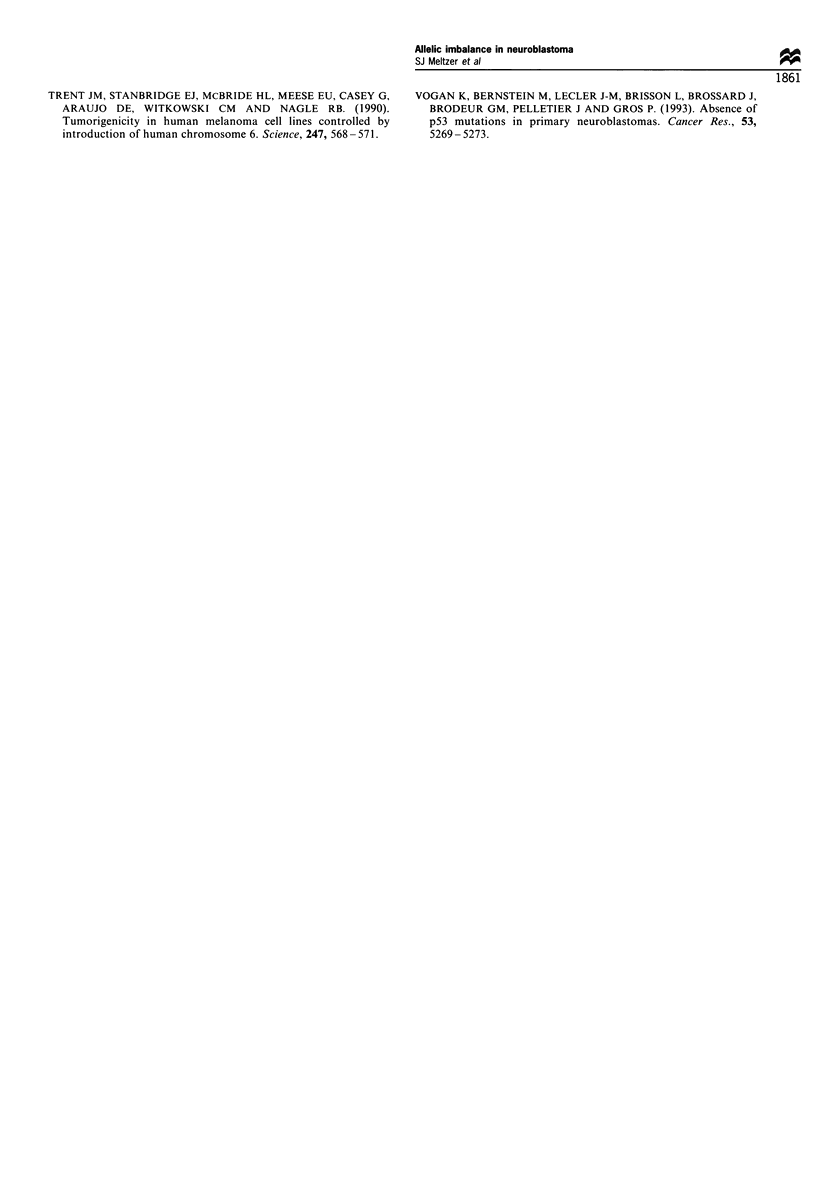


## References

[OCR_00650] Ashton-Rickardt P. G., Wyllie A. H., Bird C. C., Dunlop M. G., Steel C. M., Morris R. G., Piris J., Romanowski P., Wood R., White R. (1991). MCC, a candidate familial polyposis gene in 5q.21, shows frequent allele loss in colorectal and lung cancer.. Oncogene.

[OCR_00661] Bourhis J., De Vathaire F., Wilson G. D., Hartmann O., Terrier-Lacombe M. J., Boccon-Gibod L., McNally N. J., Lemerle J., Riou G., Bénard J. (1991). Combined analysis of DNA ploidy index and N-myc genomic content in neuroblastoma.. Cancer Res.

[OCR_00666] Boynton R. F., Blount P. L., Yin J., Brown V. L., Huang Y., Tong Y., McDaniel T., Newkirk C., Resau J. H., Raskind W. H. (1992). Loss of heterozygosity involving the APC and MCC genetic loci occurs in the majority of human esophageal cancers.. Proc Natl Acad Sci U S A.

[OCR_00682] Brodeur G. M., Sekhon G., Goldstein M. N. (1977). Chromosomal aberrations in human neuroblastomas.. Cancer.

[OCR_00687] Chen P. L., Chen Y. M., Bookstein R., Lee W. H. (1990). Genetic mechanisms of tumor suppression by the human p53 gene.. Science.

[OCR_00695] D'Amico D., Carbone D. P., Johnson B. E., Meltzer S. J., Minna J. D. (1992). Polymorphic sites within the MCC and APC loci reveal very frequent loss of heterozygosity in human small cell lung cancer.. Cancer Res.

[OCR_00706] Fearon E. R., Cho K. R., Nigro J. M., Kern S. E., Simons J. W., Ruppert J. M., Hamilton S. R., Preisinger A. C., Thomas G., Kinzler K. W. (1990). Identification of a chromosome 18q gene that is altered in colorectal cancers.. Science.

[OCR_00709] Fong C. T., Dracopoli N. C., White P. S., Merrill P. T., Griffith R. C., Housman D. E., Brodeur G. M. (1989). Loss of heterozygosity for the short arm of chromosome 1 in human neuroblastomas: correlation with N-myc amplification.. Proc Natl Acad Sci U S A.

[OCR_00716] Fong C. T., White P. S., Peterson K., Sapienza C., Cavenee W. K., Kern S. E., Vogelstein B., Cantor A. B., Look A. T., Brodeur G. M. (1992). Loss of heterozygosity for chromosomes 1 or 14 defines subsets of advanced neuroblastomas.. Cancer Res.

[OCR_00735] Hayashi Y., Kanda N., Inaba T., Hanada R., Nagahara N., Muchi H., Yamamoto K. (1989). Cytogenetic findings and prognosis in neuroblastoma with emphasis on marker chromosome 1.. Cancer.

[OCR_00743] Hayes F. A., Green A., Hustu H. O., Kumar M. (1983). Surgicopathologic staging of neuroblastoma: prognostic significance of regional lymph node metastases.. J Pediatr.

[OCR_00762] Horii A., Nakatsuru S., Miyoshi Y., Ichii S., Nagase H., Ando H., Yanagisawa A., Tsuchiya E., Kato Y., Nakamura Y. (1992). Frequent somatic mutations of the APC gene in human pancreatic cancer.. Cancer Res.

[OCR_00755] Horii A., Nakatsuru S., Miyoshi Y., Ichii S., Nagase H., Kato Y., Yanagisawa A., Nakamura Y. (1992). The APC gene, responsible for familial adenomatous polyposis, is mutated in human gastric cancer.. Cancer Res.

[OCR_00768] Imamura J., Bartram C. R., Berthold F., Harms D., Nakamura H., Koeffler H. P. (1993). Mutation of the p53 gene in neuroblastoma and its relationship with N-myc amplification.. Cancer Res.

[OCR_00782] Kaneko Y., Kanda N., Maseki N., Sakurai M., Tsuchida Y., Takeda T., Okabe I., Sakurai M. (1987). Different karyotypic patterns in early and advanced stage neuroblastomas.. Cancer Res.

[OCR_00852] Kinzler K. W., Nilbert M. C., Su L. K., Vogelstein B., Bryan T. M., Levy D. B., Smith K. J., Preisinger A. C., Hedge P., McKechnie D. (1991). Identification of FAP locus genes from chromosome 5q21.. Science.

[OCR_00801] Komuro H., Hayashi Y., Kawamura M., Hayashi K., Kaneko Y., Kamoshita S., Hanada R., Yamamoto K., Hongo T., Yamada M. (1993). Mutations of the p53 gene are involved in Ewing's sarcomas but not in neuroblastomas.. Cancer Res.

[OCR_00807] Look A. T., Hayes F. A., Nitschke R., McWilliams N. B., Green A. A. (1984). Cellular DNA content as a predictor of response to chemotherapy in infants with unresectable neuroblastoma.. N Engl J Med.

[OCR_00811] Look A. T., Hayes F. A., Shuster J. J., Douglass E. C., Castleberry R. P., Bowman L. C., Smith E. I., Brodeur G. M. (1991). Clinical relevance of tumor cell ploidy and N-myc gene amplification in childhood neuroblastoma: a Pediatric Oncology Group study.. J Clin Oncol.

[OCR_00819] MacMillan R. W., Blanc W. B., Santulli T. V. (1976). Maturation of neuroblastoma to ganglioneuroma in lymph nodes.. J Pediatr Surg.

[OCR_00837] Miyoshi Y., Nagase H., Ando H., Horii A., Ichii S., Nakatsuru S., Aoki T., Miki Y., Mori T., Nakamura Y. (1992). Somatic mutations of the APC gene in colorectal tumors: mutation cluster region in the APC gene.. Hum Mol Genet.

[OCR_00840] Nakatsuru S., Yanagisawa A., Ichii S., Tahara E., Kato Y., Nakamura Y., Horii A. (1992). Somatic mutation of the APC gene in gastric cancer: frequent mutations in very well differentiated adenocarcinoma and signet-ring cell carcinoma.. Hum Mol Genet.

[OCR_00795] Nishisho I., Nakamura Y., Miyoshi Y., Miki Y., Ando H., Horii A., Koyama K., Utsunomiya J., Baba S., Hedge P. (1991). Mutations of chromosome 5q21 genes in FAP and colorectal cancer patients.. Science.

[OCR_00858] Noble J. R., Willetts K. E., Mercer W. E., Reddel R. R. (1992). Effects of exogenous wild-type p53 on a human lung carcinoma cell line with endogenous wild-type p53.. Exp Cell Res.

[OCR_00865] Peto R., Pike M. C., Armitage P., Breslow N. E., Cox D. R., Howard S. V., Mantel N., McPherson K., Peto J., Smith P. G. (1977). Design and analysis of randomized clinical trials requiring prolonged observation of each patient. II. analysis and examples.. Br J Cancer.

[OCR_00868] Powell S. M., Zilz N., Beazer-Barclay Y., Bryan T. M., Hamilton S. R., Thibodeau S. N., Vogelstein B., Kinzler K. W. (1992). APC mutations occur early during colorectal tumorigenesis.. Nature.

[OCR_00876] Robertson G. P., Coleman A. B., Lugo T. G. (1996). Mechanisms of human melanoma cell growth and tumor suppression by chromosome 6.. Cancer Res.

[OCR_00886] Schwab M., Alitalo K., Klempnauer K. H., Varmus H. E., Bishop J. M., Gilbert F., Brodeur G., Goldstein M., Trent J. (1983). Amplified DNA with limited homology to myc cellular oncogene is shared by human neuroblastoma cell lines and a neuroblastoma tumour.. Nature.

[OCR_00891] Seeger R. C., Brodeur G. M., Sather H., Dalton A., Siegel S. E., Wong K. Y., Hammond D. (1985). Association of multiple copies of the N-myc oncogene with rapid progression of neuroblastomas.. N Engl J Med.

[OCR_00897] Shimada Y., Masayuki I. (1994). [Primary cell culture of esophageal cancer as an indicator of biological malignancy].. Hum Cell.

[OCR_00902] Srivatsan E. S., Murali V., Seeger R. C. (1991). Loss of heterozygosity for alleles on chromosomes 11q and 14q in neuroblastoma.. Prog Clin Biol Res.

[OCR_00910] Suzuki T., Yokota J., Mugishima H., Okabe I., Ookuni M., Sugimura T., Terada M. (1989). Frequent loss of heterozygosity on chromosome 14q in neuroblastoma.. Cancer Res.

[OCR_00914] The I., Murthy A. E., Hannigan G. E., Jacoby L. B., Menon A. G., Gusella J. F., Bernards A. (1993). Neurofibromatosis type 1 gene mutations in neuroblastoma.. Nat Genet.

[OCR_00922] Trent J. M., Stanbridge E. J., McBride H. L., Meese E. U., Casey G., Araujo D. E., Witkowski C. M., Nagle R. B. (1990). Tumorigenicity in human melanoma cell lines controlled by introduction of human chromosome 6.. Science.

[OCR_00928] Vogan K., Bernstein M., Leclerc J. M., Brisson L., Brossard J., Brodeur G. M., Pelletier J., Gros P. (1993). Absence of p53 gene mutations in primary neuroblastomas.. Cancer Res.

